# Single Cell Imaging of Nuclear Architecture Changes

**DOI:** 10.3389/fcell.2019.00141

**Published:** 2019-07-24

**Authors:** Rikke Brandstrup Morrish, Michael Hermes, Jeremy Metz, Nicholas Stone, Stefano Pagliara, Richard Chahwan, Francesca Palombo

**Affiliations:** ^1^School of Physics and Astronomy, University of Exeter, Exeter, United Kingdom; ^2^Living Systems Institute and School of Biosciences, University of Exeter, Exeter, United Kingdom; ^3^Institute of Experimental Immunology, University of Zurich, Zurich, Switzerland

**Keywords:** B cell, auxeticity, nuclear architecture, chromatin, infrared microscopy, microfluidics

## Abstract

The dynamic architecture of chromatin, the macromolecular complex comprised primarily of DNA and histones, is vital for eukaryotic cell growth. Chemical and conformational changes to chromatin are important markers of functional and developmental processes in cells. However, chromatin architecture regulation has not yet been fully elucidated. Therefore, novel approaches to assessing chromatin changes at the single-cell level are required. Here we report the use of FTIR imaging and microfluidic cell-stretcher chips to assess changes to chromatin architecture and its effect on the mechanical properties of the nucleus in immune cells. FTIR imaging enables label-free chemical imaging with subcellular resolution. By optimizing the FTIR methodology and coupling it with cell segmentation analysis approach, we have identified key spectral changes corresponding to changes in DNA levels and chromatin conformation at the single cell level. By further manipulating live single cells using pressure-driven microfluidics, we found that chromatin decondensation – either during general transcriptional activation or during specific immune cell maturation – can ultimately lead to nuclear auxeticity which is a new biological phenomenon recently identified. Taken together our findings demonstrate the tight and, potentially bilateral, link between extra-cellular mechanotransduction and intra-cellular nuclear architecture.

## Introduction

The nucleus of a cell is packed with strings of DNA, the genetic code that forms the basis for cell survival and function. A wide array of molecules within the nucleus participate in the control of gene expression and genomic maintenance. This is largely orchestrated through the macromolecular complex known as chromatin, which primarily consists of genomic DNA wound around histones. The structure and dynamics of chromatin which are regulated through chemical and conformational changes, are vital for normal cell functions (Venkatesh and Workman, 2015; Nair et al., 2017). We henceforth refer to this collective process as chromatin architecture.

A multitude of biochemical and molecular signaling cascades orchestrate changes to chromatin and thereby transcription patterns (MacDonald et al., 2009; Briscoe and Thérond, 2013; Yang et al., 2013; Vihervaara et al., 2018). Mechanotransduction, which enables direct force applied to the outside of a cell to impact gene transcription (Tajik et al., 2016), may also play a central role in the alterations of chromatin architecture. This signaling mechanism is thought to be mediated by Linker of Cytoskeleton and Nucleoskeleton (LINC) complexes, which have been implicated in a range of human diseases (Méjat and Misteli, 2010). Yet the mechanisms involved in the interaction between cytoskeleton and nucleus during mechanotransduction have not yet been fully elucidated (Wang et al., 2009; Fedorchak et al., 2014; Cho et al., 2017; Miroshnikova et al., 2017).

Measurements of pan-nuclear dynamics may provide further insights and novel research angles. Notably, population-based studies have been confounded by the heterogeneity observed even within “monoclonal” cell cultures. Single cell microfluidic approaches have been shown to be powerful tools for teasing out patterns that population-based assays may not reveal (Gossett et al., 2012; Otto et al., 2015; Hodgson et al., 2017). Indeed, using single cell pan-nuclear analysis, we have recently shown that chromatin decondensation can facilitate nuclear auxeticity, a mechanical property proposed to be a key element in mechanotransduction (Pagliara et al., 2014).

Pan-nuclear measurements can be achieved in many ways. DNA staining to visualize the whole nucleus provide information about shape, size, and deformability (Pagliara et al., 2014). Measurements of chromatin architecture, have generally required bespoke protein, DNA, or RNA labels (Filion et al., 2010; Deng et al., 2015; Wang et al., 2016a,b) or high-throughput DNA sequencing such as chromosome conformation capture (or 3C) and its more recent variant Hi-C, among others (Nagano et al., 2013). However, these specific tools are not without their own limitations in terms of prohibitive cost, technical difficulties, and reproducibility issues. One emerging field that may provide new tools for chromatin research, is vibrational spectroscopy-based chemical imaging. Fourier Transform Infrared (FTIR) spectroscopy, which enables label-free detection of the chemical composition and heterogeneity of a sample, has been shown to be a powerful tool for analyzing biological samples (Baker et al., 2014; Butler et al., 2016). The technique probes vibrational modes in molecules that are specific as a chemical fingerprint; through imaging or mapping approaches with a microscope, it provides molecular distributions with high spatial resolution. FTIR spectroscopy has previously been used to monitor DNA conformational changes (Whelan et al., 2011; Wood, 2016), making it a powerful biochemical tool for studying chromatin structure and conformation. Developing novel approaches to assessing chromatin changes at the single cell level would enable a more comprehensive understanding of the nuclear response.

B cells are a good model to study dynamic chromatin architecture and its links to cellular development. B cells are lymphocytes capable of producing antibodies, also called immunoglobulins (Ig), to ward off microbes and toxins (Parra et al., 2013). A tight regulation of immune cell signaling and DNA editing ensure adequate B cell maturation (Johnson et al., 2010; Vuong and Chaudhuri, 2012; Sheppard et al., 2018). As such, large-scale chemical and conformational changes to chromatin, are important markers for the development of an effective immune response (Kieffer-Kwon et al., 2017). In addition to providing a good model for studying large-scale changes in chromatin architecture, direct measurements of immune cell activation states may also be of clinical relevance. This is particularly true for label-free techniques, as they may provide further information regarding inflammatory responses *in situ*. Indeed, preliminary studies examining the immune cell state of lymphocytes and macrophages using label-free spectroscopic techniques have been published in the last couple of decades (Wood et al., 2000; Mazur et al., 2013; Hobro et al., 2016; Ichimura et al., 2016; Pavillon et al., 2018).

In this study, novel improvements to assess changes in chromatin architecture at the single cell level were explored. Chromatin decondensation was induced in B cells, through transcriptional activation or immune maturation, and evaluated using micro-transmission FTIR spectroscopic imaging and compared to untreated control cells. The sensitivity of FTIR imaging for chromatin, quantification was also examined by treating cells with cell cycle inhibitors. The effect of chromatin decondensation on nuclear morphology was assessed by an optimized microfluidics approach, which allowed for live single cell measurements. Importantly, our results reveal that some cells within the heterogeneous population experienced changes to their nuclear morphology upon chromatin decondensation. This is indicative of a differential response to mechanical stimuli dependent on the chromatin state within individual cells.

## Materials and Methods

### Cell Culture

CH12F3 B cells were cultured in HyCloneTM RPMI 1640 medium (GE Healthcare Life Sciences, SH30096.01) with 10% Fetal Bovine Serum (GibcoTM, #16140071), 5% NCTC 109 (GibcoTM, #21340039), 1% Pen-strep (GibcoTM, 10378016), 1% Glutamine (GibcoTM, #25030024), 1% Sodium Pyruvate (GibcoTM, #11360070), and 50 μM β-mercaptoethanol (GibcoTM, 31350010).

### Animals

Barrier bred 8 weeks old female C57/BL6 mice were obtained post-mortem from the Biological Services Unit, Living Systems Institute (University of Exeter), as part of the facility’s maintenance culling protocol; thereby circumventing the need for additional ethical approval. Cells were extracted from the spleen, and B cells were isolated using EasySepTM Mouse B Cell Isolation Kit (Stemcell Technologies, #19854). The isolated B cells were cultured in HyCloneTM RPMI 1640 medium (GE Healthcare Life Sciences, SH30096.01) with 10% Fetal Bovine Serum (GibcoTM, #16140071), 1% Pen-strep (GibcoTM, 10378016), 2% Glutamine (GibcoTM, #25030024), and 50 μM β-mercaptoethanol (GibcoTM, 31350010).

### Cell Treatments

For the cell cycle experiments, cells were stalled in S phase and G2/M phase, using 0.1 mM Hydroxyurea (Sigma-Aldrich, H8627-1G) for 20 h and 10 ng/ml Nocodazole (Sigma-Aldrich, M1404-2MG) for 8 h, respectively.

For the chromatin conformation experiments, cells were incubated with 10 nM Trichostatin A (Sigma-Aldrich, T8552-1MG) for 24 h to inhibit HDAC activity and thus induce hyperacetylation and decondensation of the chromatin.

For immune activation, CH12F3 cells were incubated with a cytokine cocktail (CIT) consisting of 2.5 μg/ml anti-CD40 (BD Biosciences, 553788), 10 ng/ml IL-4 (R&D Systems, 404-ML-050), and 50 ng/ml TGFβ (R&D Systems, 240-B-010). Primary B cells were incubated with 5 μg/ml Lipopolysaccharide solution (InvitrogenTM, 15526286) and 10 ng/ml IL-4. Class switch recombination was assessed by IgM to IgA switching for CH12F3 cells, and IgM to IgG1 switching for primary B cells.

### Flow Cytometry and Antibodies

Flow cytometry measurements were performed using a BD Accuri C6 Plus flow cytometer. For the cell cycle assays, cells fixed in 70% ethanol and stored at 4°C were washed in PBS and incubated in PI stain solution (PBS with 1% TritonX, 20 μg/ml RNAse A and 60 μg/ml propidium iodide) for 30 min at 37°C, placed on ice and measured.

For acetylation assays, cells fixed 1% paraformaldehyde (PFA) were stained with Histone H4ac (pan-acetyl) antibody (Active Motif, 39244) and a secondary antibody conjugated to the Alexa 647 fluorophore (Abcam, ab150067).

For class switching assays, CH12F3 fixed in 1% PFA cells were stained with FITC Anti-mouse IgA antibody (BD Biosciences, 559354) and APC Anti-mouse IgM antibody (Affymetrix eBioscience, 17-5790-82), both 1:200 dilution. Primary B cells fixed in 1% PFA were stained with FITC Rat Anti-Mouse IgM antibody (BD PharmingenTM, 553408), 1:200 dilution. Both cell types were incubated with the antibody solutions for 45 min on ice, then washed in PBS and measured.

### Sample Preparation for Micro-FTIR Imaging

Cells were washed in PBS, pelleted and re-suspended in PBS. Prior to each measurement, a 30 μl aliquot of the cell suspension was pipetted onto a calcium fluoride slide (Raman grade polished window, 20 mm diameter by 1 mm, Crystran) at a 45° angle to prevent clumping. The samples were left to rest in this position while being kept in a fridge at 4°C for 10 min, then fixed in 2% PFA for 20 min. After removal of the fixative, the samples were briefly washed in water. Cells were left to dry for minimum 36 h in a covered container. A minimum of three replicates were prepared and analyzed for each cell treatment.

### Micro-Transmission Fourier Transform Infrared (FTIR) Imaging

Micro-transmission FTIR images were collected using an Agilent imaging system consisting of a Cary 670 FTIR spectrometer, coupled to a Cary 620 IR microscope with a 0.62 NA, 15 × Cassegrain objective, and a liquid nitrogen-cooled 128 × 128 focal plane array (FPA) detector with 5.5 × 5.5 μm^2^ pixel size. The system was used in “high magnification” mode, with magnifying optics before the FPA detector, providing an additional 5× magnification corresponding to 1.1 × 1.1 μm^2^ pixel size. For each mosaic image, 2 × 2 tiles were measured with 256 scans at 4 cm^-1^ spectral resolution in the 3900–1000 cm^-1^ spectral region. A background, measured in the absence of a sample (clean area of the calcium fluoride substrate), was also measured for each sample with 512 scans. Each mosaic image had an acquisition time of approximately 50 min.

### FTIR Imaging Data Analysis

FTIR hyperspectral images are three-dimensional datasets consisting of two spatial dimensions (*x* and *y*) and one spectral dimension (*z*). Data analysis was conducted in the fingerprint region, 1800–1000 cm^-1^.

Spectral information from the cells had to be extracted from the whole FTIR images, to enable a reliable comparison between cell treatments. An image-based cell segmentation approach was applied, as it further enabled the extraction of single cell spectra from within each FTIR image. The image-based cell segmentation was conducted in Python using Otsu thresholding and watershed segmentation (van der Walt et al., 2014). An average spectrum was calculated for each cell based on the spectra within the ROI as defined by the segmentation. The spectral signatures of the samples were then compared at the single cell level, thus considering intra-sample variability and quantifying the spectral differences between cell populations.

Pre-processing of the spectra involved baseline subtraction using an offset at 1800 cm^-1^, and normalization to the Amide I peak maximum in the range 1710–1650 cm^-1^. To calculate integrated peak intensity ratios, a baseline passing through the troughs each side of the peak was drawn and the integral of the peak was calculated. The FTIR data were analyzed in R using hyperSpec (Beleites and Sergo, 2017), FTIR, gridExtra (Auguie and Antonov, 2017), and matrixStats (Bengtsson et al., 2018) software packages.

### Microfluidic Device Specifications and Preparation

A silicon mold was fabricated as previously reported (Pagliara et al., 2014). A negative of the mold was prepared through replica molding with PDMS (DOWSILTM 184 Silicone Elastomer Kit), 9:1 ratio of base to curing agent, in a small container. Once bubble-free, the PDMS was heated at 70°C for 1 h. The PDMS chip was cut to size using a scalpel, and the inlet and outlet were created with a 1.5 mm biopsy punch (Miltex, 33-31A-P/25). The chip consisted of eight square microfluidic channels with a length of 250 μm and a cross section of 8 × 8 μm^2^ connecting two large reservoirs for inlet and outlet with a depth of 20 μm.

The chip was bonded to a glass coverslip using surface ionization by oxygen plasma treatment (10 s exposure to 20W plasma power in 1 mbar of air, Diener Royal Oak). The chip was functionalized with 1 mg/ml BSA and incubated at 37°C for 1 h (Bamford et al., 2017).

### Microfluidic Experiment

Cells were spun down (300 g × 5 min) and resuspended in 0.5 μg/ml Hoechst 33342 (ThermoFisher Scientific, H3570) for CH12F3 cells and 5 μg/ml Hoechst 33342 for primary B cells. Following incubation at 37°C for 20 min, the cells were spun down again, washed in PBS and resuspended in PBS, 50 μM β-mercaptoethanol (GibcoTM, 31350010), 15% OptiPrep (Sigma Aldrich, D1556-250ML) for CH12F3 cells and PBS, 50 μM β-mercaptoethanol, 10% OptiPrep for primary B cells.

Cells were flowed into the microfluidic chip at a constant applied pressure of 1 mbar through Portex tubing PE 0.86 × 0.33 mm B × W (Scientific Laboratory Supplies, TUB26668) using a Fluigent pump. This resulted in a cell average velocity of 0.55 ± 0.06 mm/s that was constant during channel translocation. The microfluidic chip was mounted on an epifluorescence inverted microscope (Olympus IX73) equipped with a 40×, 0.95 N.A. objective and a sCMOS camera (Andor Zyla 4.2) used at a frame rate of 30 fps. A minimum of three replicates were prepared and analyzed for each cell treatment.

### Nucleus Deformation Data Analysis

Image analysis of nucleus deformation was performed using Python, in particular the modules scikit-image (van der Walt et al., 2014), imageio, numpy (Oliphant, 2006), and SciPy (Jones et al., 2001). Cell nuclei were identified using li thresholding and tracked between frames. Fragmented trajectories were stitched together manually. Properties, including minimum and maximum axis, were saved for each nucleus in each frame. These were used to calculate average nuclei size within region 1 (before channels) and region 2 (within channels), as well as transverse strain, S_T_ (S_T_ = (minimum axis_in channel_ – minimum axis_before channel_)/minimum axis_before channel_) and axial strain, S_A_ (S_A_ = (maximum axis_in channel_ – maximum axis_before channel_)/maximum axis_before channel_).

### Statistical Analyses

Unless otherwise stated, unpaired *t*-tests were used, on at least 3 biological replicates, to determine the statistical significance of the difference in means between groups. This was done for both the DNA-to-protein FTIR peak ratios and the calculated nuclear transverse strain (S_T_) for small nuclei from control, activated and TSA treated cells.

## Results

### Novel Optimization of Micro-FTIR Imaging for Single Cell Analysis

**FIGURE 1:**
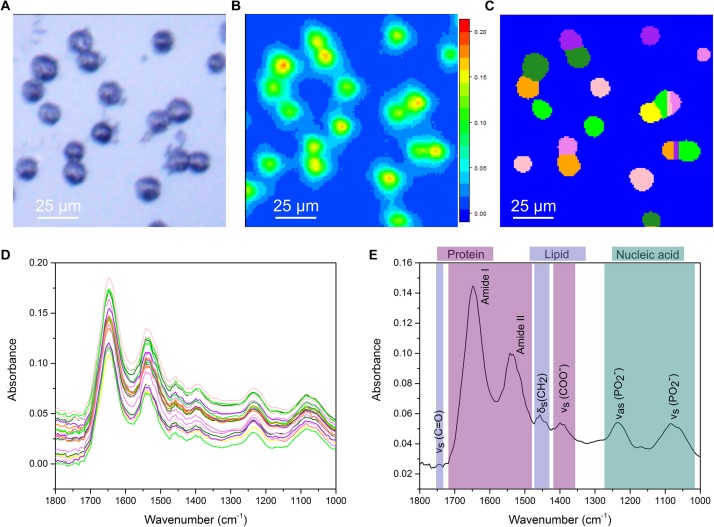
Single cell analysis for FTIR imaging. **(A)** White-light image measured in reflection mode. **(B)** Chemical image based on 1625–1675 cm^-1^. **(C)** Output of the cell segmentation of the data in **(B)**. This image segmentation is used to extract an average spectrum from each cell. **(D)** Extracted single cell spectra. The colors correspond to the colors in **(C)**. **(E)** A representative FTIR spectrum of a single cell with key vibrational modes indicated by their corresponding peaks. Peaks primarily associated with protein, lipid and nucleic acids are color coded.

Previous work, from our group and others, has shown FTIR imaging of biological samples (Nallala et al., 2016; Perez-Guaita et al., 2016; Shinzawa et al., 2017) but efficiently extracting average single cell spectra from large FTIR images has remained problematic. Smaller FTIR images of single cells, or selection of areas of interest within larger images post-acquisition, while able to circumvent this, are time consuming approaches. We addressed this using a combination of experimental and computational optimizations. The photomicrograph of an untreated CH12F3 cell sample acquired using a 15× Cassegrain objective is shown in Figure 1A. Cells appear as dark ovoids fairly homogeneously distributed on the transparent microscope slide. A chemical image based on the intensity (peak maximum) of the Amide I band, essentially C = O stretching of the peptide group, in the range 1675–1625 cm^-1^ is presented in Figure 1B. Note that FTIR images are pseudo-color images where the high absorbance, represented by red color, corresponds to high concentration of a particular chemical species, in this case proteins, whilst low absorbance represented by blue color corresponds to low concentration or absence of that species. Here, cells are identified by the high protein signal (Amide I) corresponding to red-to-green areas in Figure 1B. Image segmentation analysis provided separation of small clusters of cells into single cells (Figure 1C), so that an average spectrum per single cell was extracted (Figure 1D). This enabled downstream analysis of the spectral signatures of differentially treated cell populations at the single cell level. Spectral analysis was performed in the “fingerprint” region (1800–1000 cm^-1^), which contains characteristic signals from proteins, lipids, and nucleic acids. Figure 1E illustrates the assignment of the main absorption peaks in this region.

### DNA Quantity Measurements Using FTIR Imaging During Cell Cycle Progression

**FIGURE 2:**
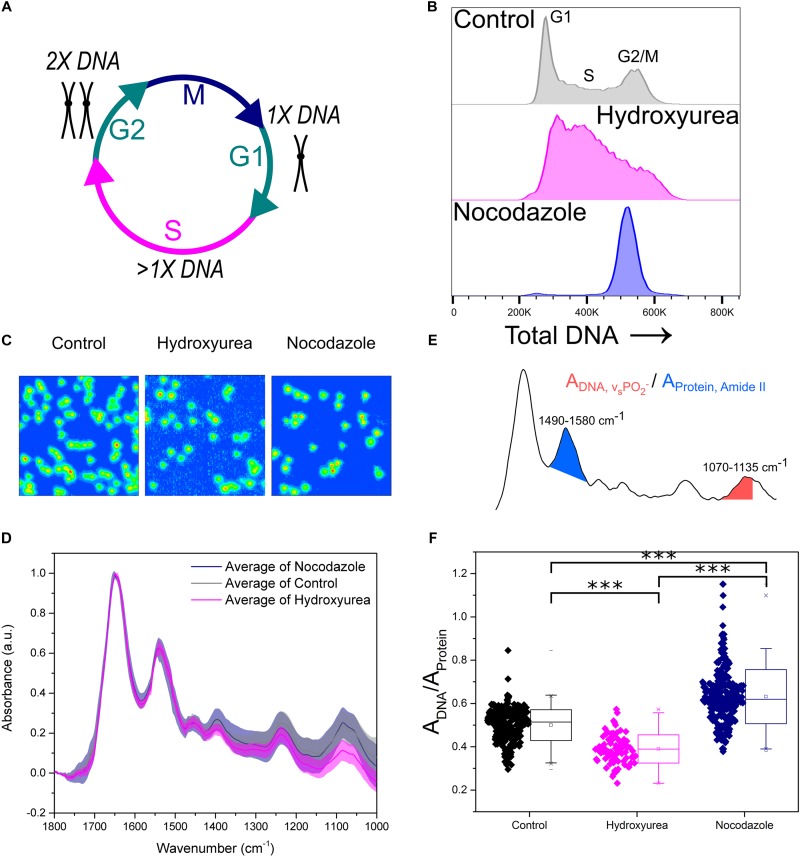
Key FTIR spectral signatures associated with intracellular DNA levels identified. **(A)** Schematic of the cell cycle. Cells go from G1 phase with 1× DNA, through S phase where the DNA is replicated, to G2 phase with 2× DNA. Following this the cells enter M phase where they undergo mitosis and divide into two daughter cells. **(B)** Flow cytometry assessment of intracellular DNA content of cell samples: Control, Hydroxyurea treated and Nocodazole treated. DNA is stained by the fluorescent marker propidium iodide. **(C)** FTIR images of cell samples Control, Hydroxyurea treated and Nocodazole treated. **(D)** Average single cell spectrum for each cell treatment. Standard deviation is marked as a shaded area. **(E)** Representative spectrum indicating the peak ratios used for the single cell analysis in **(F)**. **(F)** DNA-to-Protein peak ratio showing the spectral changes associated with differing levels of intracellular DNA at the single cell level. A *t*-test gave a statistically significant difference between samples (^∗∗∗^*P* ≤ 0.001).

Peak absorbance in the FTIR spectrum is related to the concentration of a particular chemical species (Beer-Lambert law). Here, we validated the application of micro-FTIR imaging to detect changes in intracellular DNA content and to investigate the effect of cell cycle progression on the molecular properties derived from FTIR spectroscopy. The cell cycle is the process used by cells to couple DNA duplication (S-phase) with cell division (M-phase). These two phases are interjected by two growth phases known as G1 and G2 phase, respectively (Figure 2A). FTIR images of cell populations stalled in early S phase (1× DNA content) and G2/M phase (2× DNA content) were therefore compared with untreated control samples.

Hydroxyurea and Nocodazole are two distinct chemical drugs utilized to arrest cells at specific phases of the cell cycle. Treatments with these stall cells in early S-phase or at the G2/M transition phase, respectively (Figure 2A,B). The effect of each drug on CH12F3 cells was assessed by propidium iodide (PI) staining and flow cytometry (Figure 2B). An increase in PI signal correlates with an increase in DNA content (Crissman and Steinkamp, 1973). The control cells are distributed across the different cell cycle phases, starting from an initial peak representing cells in G1 phase (1× DNA content), spanning the increase in DNA content during S phase (>1× DNA content), and ending in a peak for cells in G2/M phase (2× DNA content). Cells treated with Hydroxyurea are stalled in early S phase, which is denoted by a large main peak at low PI signal. In contrast, cells treated with Nocodazole give rise to a single peak at high PI signal, indicating stalling in G2/M phase.

Figure 2C shows FTIR images of representative samples for each cell group. Average FTIR spectra calculated from single cell spectra within each group show differences (Figure 2D), especially in the phosphate symmetric stretching (ν_s_ PO_2_^-^) peak at 1070–1035 cm^-1^ which is due to intracellular DNA. We used the integrated intensity ratio of ν_s_ PO_2_^-^ to Amide II peak (1580–1490 cm^-1^, as opposed to the Amide I which may contain contribution from the water bending mode) to quantify changes in DNA to protein ratio at the single cell level (Figure 2E). The DNA-to-protein ratios were significantly different between control, S and G2/M phase cell populations. The observed differences reflected changes in DNA content, with lower values for the S phase stalled cells (hydroxyurea treatment, pink) compared to control cells, and higher values for the G2/M phase stalled cells (nocodazole treatment, navy).

The correlation between the DNA-to-protein ratio and the intracellular DNA content is unsurprising, as a change in concentration leads to a change in absorbance. The FTIR spectral changes are potentially caused by more than a simple decrease or increase in intracellular DNA. Significant changes to DNA environment and structure, which are especially apparent for chromosome condensation during G2/M phase, likely also influence the absorbance, as this would incur changes to local densities and the extinction coefficient. We therefore decided to test whether FTIR imaging can detect changes in DNA structure and environment, when these are independent from cell cycle phase and thus intracellular DNA content.

### DNA Quality Measurements Using FTIR Imaging of Chromatin Changes

**FIGURE 3:**
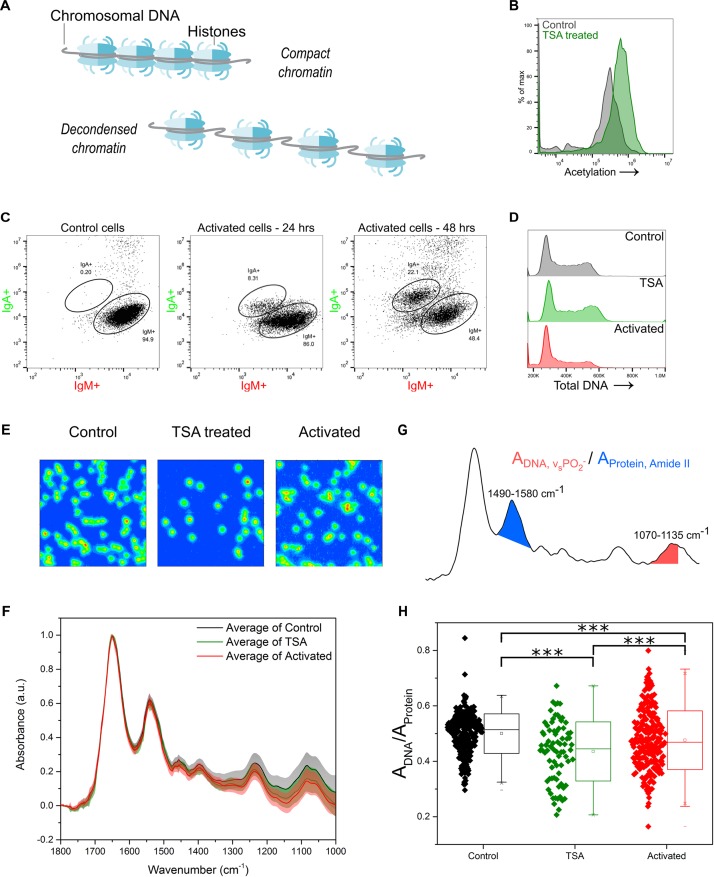
Chromatin changes can be assessed using FTIR imaging. **(A)** Cartoon representation of chromatin, in compact and decondensed state. **(B)** Verification of the effect of TSA treatment, as seen by increased acetylation levels in TSA treated cells compared to control cells. Intracellular acetylation level labeled by an anti-pan acetyl antibody and measured by flow cytometry. **(C)** CSR measured as verification of immune activation of B cells through CIT treatment. A subpopulation, increasing in size over 48 h, switches from IgM to IgA expression. **(D)** Cell cycle phase distribution for TSA treated or activated cells compared to control cells. DNA is stained by the fluorescent marker propidium iodide. **(E)** FTIR images of cell samples Control, TSA treated and Activated (CIT treated). **(F)** Average single cell spectrum for each cell treatment. Standard deviation is marked as a shaded area. **(G)** Representative spectrum indicating the peak ratios used for the single cell analysis in **(H)**. **(H)** DNA-to-Protein peak ratio showing the spectral changes associated with changes to chromatin state at the single cell level. A *t*-test gave a statistically significant difference between samples (^∗∗∗^*P* ≤ 0.001).

DNA rarely exists in isolation within the cell nucleus. Indeed, the macromolecular complex known as chromatin, consists primarily of genomic DNA wound around a complex of histone proteins (Figure 3A). Although DNA quantity does not vary during the G1 or G2 growth phases of the cell the chromatin fibers do still respond to intra- or extra-cellular stimulations which can alter the quality and architecture of the chromatin complex.

Trichostatin A (TSA) is an inhibitor of histone deacetylases which primarily function as transcriptional repressors. Treating the CH12F3 cells with TSA, causes hyperacetylation of the histones which results in chromatin decondensation. This increase in acetylation was verified by flow cytometry, using an antibody against pan-acetylation (Figure 3B). Chromatin decondensation is an essential intermediate in a number of cell processes, including immune activation of B cells, where it facilitates the increased transcription associated with activated B cells (Fowler et al., 2015; Zhang et al., 2016). TSA treatment of cells can therefore mimic the chromatin modifications observed during immune cell activation. Indeed, lymphocyte stimulation has previously been shown to induce an increase in acetylation of the chromatin (Pogo et al., 1966; Kouzine et al., 2013; Rawlings et al., 2011). In addition, secondary antibody diversification, a result of activation of B cells via antigen binding, can be initiated in CH12F3 cells through addition of a cytokine cocktail (CIT) consisting of IL-4, TBF-β, and anti-CD40. The resultant class switch recombination, where a subset of the cells undergoes DNA recombination leading to changes to the expressed antibody constant region, was assessed using flow cytometry and antibodies against the Ig isotypes IgM and IgA (Figure 3C).

The effect of chromatin decondensation, induced through TSA treatment or CIT mediated immune activation, was investigated here. Cell cycle phase distribution was assessed as before by flow cytometry. Importantly, neither TSA nor CIT treatment resulted in changes to overall DNA content (Figure 3D). Therefore, any differences observed in FTIR spectra would derive from “qualitative” chemical and conformational changes to the chromatin, rather than from changes to overall DNA “quantity” (Supplementary Figure S1).

FTIR images and spectral analysis for this study are presented in Figure 3E–G. FTIR spectra from TSA and CIT treated cells were found to vary with respect to those of control cells (Figure 3F), especially in the phosphate stretching peaks. The single cell DNA-to-protein ratio shows a statistically significant decrease for both TSA and CIT treated cells when compared to control cells (Figure 3G). Although a significant difference was found between TSA and CIT treated cells, the similar response compared to control cells is consistent with the similar chromatin response expected for the two treatments. Furthermore, it supports the consensus that while only a subset of the cells successfully switches Ig isotype from IgM to IgA, all cells are initially activated by the addition of the CIT.

### Nuclear Architecture Response

**FIGURE 4:**
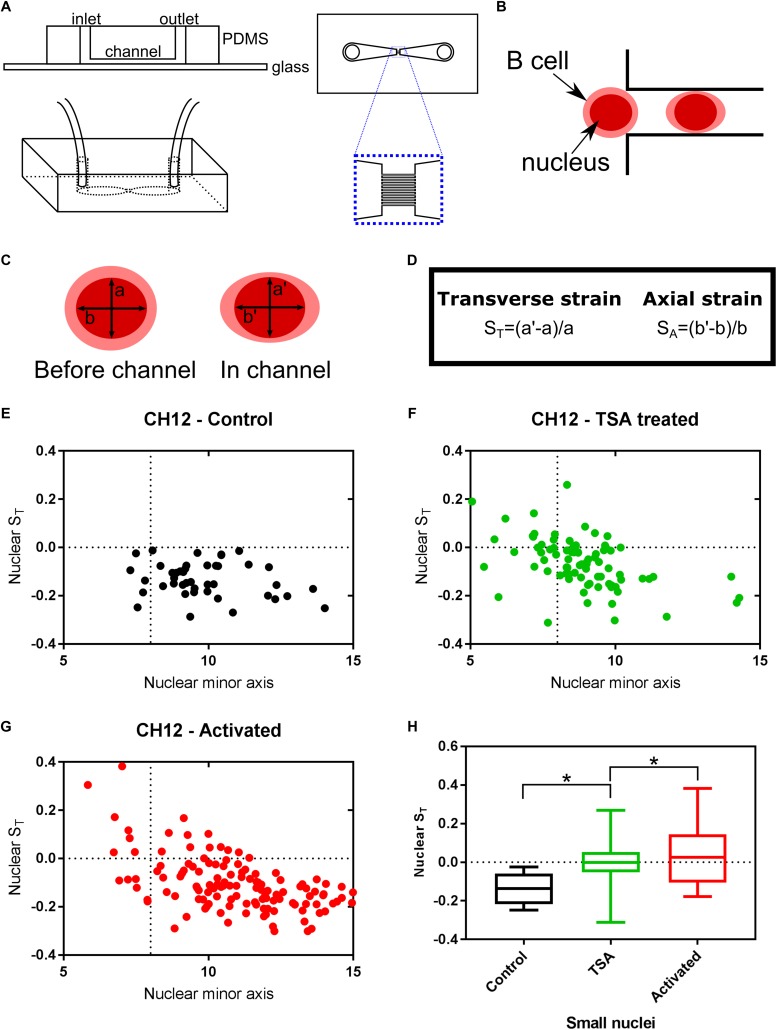
Chromatin decondensation is correlated with auxetic nuclei of B cells. Cells visualized with the nuclear stain Hoechst 33342 were imaged translocating channels of a microfluidic chip. **(A)** Schematic of the microfluidic chip used to stretch cells. **(B)** Schematic of a cell moving through the channel. The cell is larger than the channel, but the nucleus is smaller. **(C)** The axes measured for each nucleus before the channel and in the channel. **(D)** Transverse and Axial strain calculations based on the axes seen in **(C)**. **(E)** Transverse strain versus nuclear minor axis for Control cells. **(F)** Transverse strain versus nuclear minor axis for TSA treated cells. **(G)** Transverse strain versus nuclear minor axis for Activated (CIT treated) cells. **(H)** Quantification of the change in transverse strain for small nuclei (<8μm), i.e., nuclei smaller than the channel width. A *t*-test gave a statistically significant difference between samples (^∗^*P* ≤ 0.05).

Just as DNA does not exist in isolation within the nucleus, chromatin is not an isolated entity either. The interaction between chromatin, the nuclear envelope and the cytoskeleton has been shown to alter transcription-associated responses through mechanotransduction (Lammerding et al., 2004; Iskratsch et al., 2014; Tajik et al., 2016; Kirby and Lammerding, 2018). We have previously proposed auxeticity of the nucleus to be an element in these signaling systems (Pagliara et al., 2014). Auxeticity is the term used to describe materials with a negative Poisson’s ratio. This means that auxetic materials exhibit a cross-sectional expansion when stretched and a cross-sectional contraction when compressed. Most materials, on the contrary, are non-auxetic, becoming thinner when stretched and therefore having a positive Poisson’s ratio. In order to investigate possible auxetic properties in B cells, we utilized a microfluidic device consisting of two large chambers for the fast delivery and collection of Hoechst stained live cells, connected via an array of channels with a square cross section of size 8 × 8 μm^2^, thus smaller than the typical size of the B cells under investigation (Figure 4A,B). These channels enable stretching stresses to be imposed upon the cell nucleus caused by cytoskeletal strain when the cell is confined in the channel. Moreover, nuclei larger than the channel cross section also experience a transverse compression. We used this assay to examine the effects of TSA and CIT treatment of CH12F3 cells on nuclear deformation in response to applied mechanical stress. The nuclear deformation in response to the pressure to the outside of the cell was determined using a nuclear stain and an epifluorescence inverted microscope as described in the methods section and shown in Supplementary Figure S3.

The transverse strain, quantifying the deformation of the nucleus due to the channel confinement in the direction perpendicular to the channel’s longitudinal axis, was used as a proxy for nuclear auxeticity (Pagliara et al., 2014; Figure 4C,D). By tracking single cells, as they moved through the channels, this axis size was determined for each cell before they enter the channel (a) and during the translocation through the channel (a’). The transverse strain was calculated from these values for each cell. A positive transverse strain indicates auxetic properties.

Untreated CH12F3 cells (Control) did not exhibit auxetic properties during the translocation through the channels. Instead their nuclei became thinner as indicated by the negative transverse strain (Figure 4E). In contrast, both TSA and CIT treated cells contained a subpopulation of cells that had positive transverse strains (Figure 4F,G). As expected, these cells were primarily found within the group of cells with nuclei that were smaller than the channel width and thus could expand in volume in the channel exhibiting an auxetic behavior. The difference in transverse strain values for small nuclei cells between treated and untreated cells were found to be statistically significant (Figure 4H). Finally, we did not find any correlation between auxeticity and cell speed.

### Primary B Cells Largely Follow the Same Patterns

**FIGURE 5:**
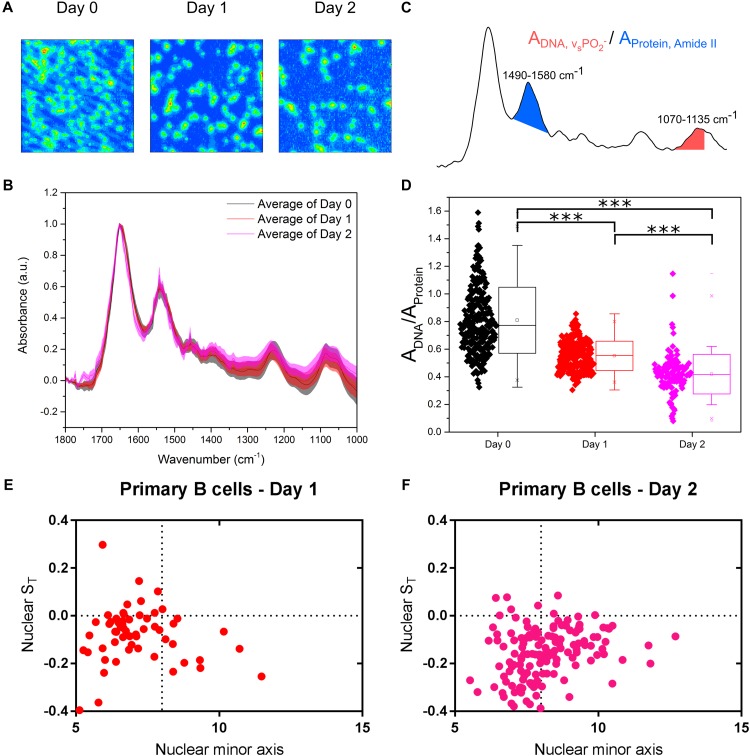
Primary murine B cells reveal are more complex picture. **(A)** FTIR images of cell samples fixed at immediately after isolation from mouse spleen (Day 0) and after being immune activated and cultured for 24 and 48 h (Days 1 and 2). **(B)** Average single cell spectrum for each cell treatment. Standard deviation is marked as a shaded area. **(C)** Representative spectrum indicating the peak ratios used for the single cell analysis in **(D)**. **(D)** DNA-to-Protein peak ratio showing the spectral changes associated with changes to DNA and chromatin in primary B cells at the single cell level. A *t*-test gave a statistically significant difference between samples (^∗∗∗^*P* ≤ 0.001). **(E)** Transverse strain versus nuclear minor axis for “Day 1” primary B cells. **(F)** Transverse strain versus nuclear minor axis for “Day 2” primary B cells.

Undoubtedly, cell lines are valuable tractable models to study a wide range of biological processes. However, primary cells offer an even more physiological outlook at what is happening in animal cells *in vivo*. In fact, the results between cell lines and primary cells are not always in agreement because of the inherent differences between them. These differences, quantified by diverging transcriptional and proteomic profiles of a number of cell types (Sandberg and Ernberg, 2005; Alge et al., 2006; Olsavsky et al., 2007; Pan et al., 2009), commonly relate to cell cycle, proliferation and metabolic processes. We therefore isolated primary B cells from mouse spleens and we immune activated these cells in culture. FTIR imaging (Figure 5A) was used to assess chromatin changes, and microfluidic chips were used to investigate their nuclear deformation. The primary B cells were measured immediately after isolation (Day 0) and then at two timepoints (Days 1 and 2) following culture in activating B cell medium containing Lipopolysaccharide solution (LPS) and Interleukin-4 (IL-4). LPS and IL-4 induce class switching in primary B cells, from the IgM to the IgG1 isotype, which was monitored by flow cytometry (Supplementary Figure S2).

Comparison of the FTIR spectra of primary B cells between Days 0, 1, and 2 showed the expected differences (Figure 5B). Single cell DNA-to-protein ratios made this more evident, with clear reductions at Day 1 from Day 0, and further reductions at Day 2 (Figure 5C). Whilst the peak ratio values (Figure 5C) vary from those of the CH12F3 the changes between Days 0, 1, and 2 for the primary B cells follow the same expected pattern we observed for CH12F3 activation with CIT (Figure 3H); thereby, strengthening the correlation between chromatin decondensation and reduction in this peak ratio.

In terms of auxetic properties of primary B cells, the pattern appears less obvious. Some cells with small nuclei do indeed exhibit auxetic properties for both Days 1 and 2 primary B cells, which is in line with what we observed for activated CH12F3 cells (Figure 5D–F). However, many other primary cells do not. We believe the reason for that is technical rather than biological.

Primary B cells differ from CH12F3 D0 cells in size (Supplementary Figures S2A,B) and cell cycle phase distribution (Supplementary Figures S2C,D). That is primarily because D0 primary B cells isolated from a tightly packed spleen tissue require few hours to adjust to growth in medium *ex vivo*. Indeed, the cells increase in size after isolation; D0 cells are smaller than D1 cells, which in turn are smaller than D2 cells (Supplementary Figure S2A). These differences in size, although less pronounced, are also reflected in the size of the nucleus (Supplementary Figure S2B) which complicates our nuclear auxeticity measurements because the stretching microchannels are too big to induce the mechanostranduction signaling needed to elicit nuclear auxeticity. That is why we could only observe the nuclear auxeticity in the small portion of primary B cells that were bigger in size. Cell cycle phase distribution is another aspect that differentiates primary B cells from the cell line. D0 primary B cells are primarily in a quiescent G0/G1 state until they are activated by antigen (Tomura et al., 2013). The proportion of cells in S and G2/M phase increases as the cells adjust in to growth in medium (Supplementary Figures S2C,D). But we do not believe that these minor changes in cell cycle distribution can account for FTIR read difference between D0, D1, and D2 (Figure 5D). In fact, the effect of primary B cell maturation is so pronounced that we see a decrease in the FTIR peak ratio rather than an increase if the cell cycle is having a significant effect as shown in Figure 2F. Interestingly B cell activation in primary cells is known to peak at 48 h which is also the timepoint we see the biggest effect in our FTIR readout, which further supports our conclusion.

## Discussion

A broad understanding of chromatin architecture dynamics is arguably one of the main hurdles to better understand cell function at the epigenomic level. Despite the numerous assays available to measure chromatin architecture, none so far can capture the full breadth of chromatin dynamics at the single cell level. That is why we optimized the FTIR imaging technique to visualize single-cell chromatin changes during immune cell development. Immune B cells were chosen because of their highly tractable properties in terms of proliferation, maturation, manipulation, and quantification. We therefore assessed changes to intracellular DNA quantity and quality of single cells using a label-free chemically specific method based on FTIR spectroscopic imaging. We assessed the capability of FTIR imaging to detect DNA changes by stalling cells in early S phase and G2/M phase. Focusing on DNA-to-protein peak ratios our results show that stalled S and G2/M phase cells appeared significantly different from untreated control cells. The differences in this peak ratio between the differently treated cell populations followed the expected pattern considering the relationship between molecular density and FTIR absorbance. Cells stalled in G2/M phase, where cells contain twofold DNA content (2×) showed the highest intensity peak, while cells stalled in early phase, where cells only contain onefold DNA content (1×) had the lowest. The untreated control cells, which contained cells in all phases were in between the two.

DNA quality, or chromatin chemical and conformational state, was also assessed using FTIR imaging, with the analysis focusing on the same peak ratio. TSA and CIT treatment of cells induce global transcriptional activation and specific B cell maturation, respectively. We observed that both treatments resulted in chromatin decondensation, and that this was associated with a decrease in the DNA-to-protein peak ratio. Assessment of cell cycle phase distribution demonstrated that these spectral changes were not originating from changes in intracellular DNA content, but rather changes to the chromatin architecture. This trend was further reproduced in primary B cells. As the chromatin unravels passing from a compact to an open and less ordered structure, the density of its components changes, with histone octamers essentially retaining their structure whilst the overall chromatin structure unfolds in terms of histone octamer spacing and decrease in DNA compaction. Due to the differential density, the DNA-to-protein ratio is a viable signature of chromatin structure and conformation. It is therefore reasonable to conclude that chromatin decondensation can be assessed through this peak ratio. Combining this technique with microfluidic devices to enable live cell imaging has the potential to provide a label-free method of assessing cell health or developmental state.

The correlation between chromatin decondensation and nuclear mechanostransduction was further assessed using epifluorescence and microfluidics, examining the recently identified cellular property termed nuclear auxeticity (Pagliara et al., 2014). Interestingly, both TSA treatment and the CIT activation of B cells resulted in similar auxetic responses in our setup but not in mock controls. Our data show, for the first time that cellular maturation can elicit a mechanical conformational change within the overall cellular structure. Our data also suggest that nuclear auxeticity correlates with actively decondensing chromatin; which also coincides with the stem cell reprogramming properties we have previously identified (Pagliara et al., 2014).

Despite the lack of a conclusive biological mechanisms for our observations, we could postulate that the link between the perturbations in chromatin architecture we initiated and the observed nuclear auxeticity could either be due to: (1) a passive conformational change whereby the chromatin decondensation created by transcriptional activation or B cell maturation causes an increase in nuclear volume via increase in fluid influx; and/or (2) an active conformational change resulting from the tight crosstalk between cytoskeletal and chromatin architecture which has been well documented so far (Crisp and Burke, 2008; Alam et al., 2015; Alam et al., 2016; Tajik et al., 2016; Miroshnikova et al., 2017; Kirby and Lammerding, 2018; Uzer et al., 2018). It has been shown that increased nuclear membrane tension can alter nuclear pore complex (NPC) permeability. NPCs are known to undergo conformational changes that constrict or dilate the NPC in response to mechanical forces (Solmaz et al., 2013; Chug et al., 2015; Stuwe et al., 2015). Most of these signaling cascades are mediated by nuclear membrane proteins known as lamins. Lamin-A/C-deficient and -mutant cells fail to adequately trigger mechanoresponsive genes following mechanical stimuli (Lammerding et al., 2004; Cupesi et al., 2010; Bertrand et al., 2014). It would be interesting in the future to test our experimental setup in Lamin deficient cells.

We could further postulate that this potential cytoskeletal/nuclear crosstalk is: (i) bilateral, whereby mechanical signals from the nuclear architecture can also affect cytoskeletal dynamics, and (ii) regulated, whereby these crosslinking signals are more pronounced during important cellular transitions and less acute during static cellular growth. Our working rationale is that much like mechanotransduction mechanisms at the cell surface could lead to substantial conformational changes in chromatin and ultimately transcriptional control; so too could chromatin conformational changes lead to alteration in overall mechanical properties of the cell. Interestingly, studies have previously shown that lymphocytes could be susceptible to mechanical signaling (Wan et al., 2015; Huse, 2017), but a direct mechanical signaling from the surface of the cells to the chromatin have not yet been shown. This regulated and bilateral crosstalk we are hereby proposing could potentially mediate a feedback loop for mechanical transduction signaling that ensures adequate cell development and function.

And while a subset of our B cell nuclei showed auxetic properties, many did not. We could attribute such discrepancy to (i) the dormant G0 state of non-activated primary B cell; (ii) technical difficulties; and/or (iii) inherent robustness of primary cells against mechanical manipulation. Non-immunized B cell splenocytes are known to exist in a quiescent G0 states which drastically decreases the active properties of these cells (Tomura et al., 2013) thereby hindering the manifestation of adequate auxetic properties in the timeframe we studied. Furthermore, for purposes of consistency, we have conducted all our auxetic measurements in microfluidic chip equipped with constriction channels with a cross section of 8 × 8 μm^2^. However, primary B cells were slightly smaller than B cells from our cell line possibly limiting our ability to propagate adequate mechanical pressure on the cell surface. In addition, it has been shown that primary cells are naturally more robust in various signaling cascades due to the inherent redundancies that are retained in primary cells and which tend to be lost in cancer cell lines used in culture (Blackford and Jackson, 2017; Brown et al., 2017). We do not think this is a strong contributing factor though because we do indeed observe some auxetic primary B cell nuclei, presumably because they had a large enough size to undergo the mechanotransduction process in our microfluidics chip. Taken together, our FTIR and nuclear auxeticity data do hold true in both B cell lines and primary B cells, however, the likelihood of technical limitations inherent to primary B cells should be taken into consideration for the nuclear auxeticity measurements.

In summary, we have optimized a novel tool for assessing chromatin architecture at the single cell level. By assessing both biochemical and mechanical changes for the same cell treatments, we have correlated changes in chromatin architecture with nuclear auxeticity. Our data corroborates previous work done in stem cells and raises the question if nuclear auxeticity is a general feature of cellular development. If so, this suggests that nuclear auxeticity could be a general phenomenon of active global cellular transcription and/or a property of cellular development whereby the nuclear architecture develops a specific property to accommodate the extensive perturbations and modifications taking place throughout the chromatin. It would be interesting though in the future to identify mechanisms that interfere with nuclear auxeticity to study the effect of its blockade on normal cellular development and differentiation. Furthermore, mechanotransduction has been implicated in key cellular developmental processes, including the immune system (González-Granado et al., 2014; Wan et al., 2015), and is speculated to account for lack of efficiency in cellular differentiation or trans-differentiation *ex vivo*. Indeed, the use of B cell organoids, 3D structures mimicking the mechanical forces displayed in lymphoid tissues *in vivo*, has been shown to greatly enhance antibody diversification in B cells through immune activation, indicating a role of mechanical and structural properties (Purwada et al., 2015). Understanding the role of auxeticity and the potential feedback loop between the nucleus and cytoskeleton, could further advance our understanding of the role of chromatin conformation and mechanical forces – and the interplay between the two – in cell development.

Finally, we wish to note that our observations need not be confined to B cell biology. We predict that many of our observations herein could be applicable to other cell types undergoing differentiation, re-differentiation, or trans-differentiation whereby fundamental and global chromatin changes are required. It also has not escaped our attention that cancer cells have been known to take advantage of trans-differentiation to maintain their survival and/or malignancy according to the cancer stem cell hypothesis (Gupta et al., 2011; Kreso and Dick, 2014). It would be interesting if our assays could be tested in the future as a diagnostic tool for tumor progression or severity at the single tumor cell level.

## Data Availability

The raw data supporting the conclusions of this manuscript will be made available by the authors, without undue reservation, to any qualified researcher.

## Author Contributions

FP, RC, SP, and NS conceived and designed the experiments. RM and SP constructed the microfluidic chip. RM performed the experiments. RM, MH, and JM analyzed the data. RM wrote the manuscript with inputs from the other authors.

## Conflict of Interest Statement

The authors declare that the research was conducted in the absence of any commercial or financial relationships that could be construed as a potential conflict of interest.
