# Supplementary material for: Single Cell Imaging of Nuclear Architecture Changes
Source: Front Cell Dev Biol. 2019 Jul 24;7:141. doi: 10.3389/fcell.2019.00141 (PMC6668442; doi:10.3389/fcell.2019.00141)
Supplement: Supplementary file 1 [file Data_Sheet_1.docx]

Single cell imaging of nuclear architecture changes

Rikke Brandstrup Morrish^1,2^, Michael Hermes^1^, Jeremy Metz^2^, Nicholas Stone^1^, Stefano Pagliara^2^*, Richard Chahwan^3^*, Francesca Palombo^1^*

^1^School of Physics and Astronomy, University of Exeter, Exeter EX4 4QL, UK.

^2^Living Systems Institute, University of Exeter, Exeter EX4 4QD, UK.

^3^Institute of Experimental Immunology, University of Zurich, 8057 Zurich, CH.

*Equally contributing authors and to whom correspondence should be addressed:

Francesca Palombo ([f.palombo@exeter.ac.uk](mailto:f.palombo@exeter.ac.uk))

Richard Chahwan ([richard.chahwan@uzh.ch](mailto:richard.chahwan@uzh.ch))

Stefano Pagliara ([s.pagliara@exeter.ac.uk](mailto:s.pagliara@exeter.ac.uk))

Supplementary Materials

**Second derivative analysis of FTIR spectra**

The second derivative of the average single cell FTIR spectrum of all samples was calculated to investigate changes in Amide I sub-peak positions between cell treatments. Only minor changes were observed, suggesting that protein secondary structure (24, 76) is essentially preserved. This rules out major conformational changes to the protein component of chromatin and the cell overall.

Fig. S1 shows the second derivative for the Control, TSA treated, and CIT treated cells. The main variation in peak position was found in the Amide I (Fig. S1C) and Amide II (Fig. S1D) regions of the spectra. The Amide I band, which is attributed to the carbonyl stretching of the peptide group, has a doublet at ~1654 and ~1640 cm^–1^ associated with α-helix and β-sheet structures, respectively (24, 76). Peak shifts between cell treatments were not significant.

**Primary B cells**

There are inherent differences between cultured cell lines and primary cells, as discussed in the main text. Therefore, it was important to characterise a number of properties of the isolated cells through the different timepoints. Beyond checking that the cells were indeed undergoing class switch recombination in response to the IL-4 and LPS treatment (Fig. S2E), changes to size (Fig. S2A-B) and cell cycle phase distribution (Fig. S2C-D) were also monitored.

The size of the cells increased going from day 0 (D0) to day 1 (D1) and further to day 2 (D2), as measured by flow cytometry (Fig. S2A). The nuclear size also increased, although to a lower degree. This was measured by staining the cell nuclei with the DNA dye Hoechst and imaging them in microfluidic chips (Fig. S2B). The difference in size influenced the additional flow cytometry measurements, as the cells stained to different levels. This was the case for both propidium iodide staining of the DNA and staining with fluorescently tagged anti-IgM antibody, evident by the changes in signal intensity between timepoints (Fig. S2C). It was therefore not possible to compare cells from different timepoints directly using gates based on the same signal intensities. However, relative changes in cell cycle phase distribution and number of IgM+/IgM- cells were still possible and therefore used to compare the different timepoints.

Immediately post isolation from mice spleens (D0), almost all of the primary B cells were found to be G0 stalled (1× DNA). For the following timepoints (D1 and D2), the proportion of cells in S (>1× DNA) and G2/M (2× DNA) phase increased (Fig. S2C-D). The growth medium, IL-4 and LPS stimulate the cells to proliferate, so the observed change in cell cycle phase distribution was expected.

The addition of IL-4 and LPS also stimulate cells to undergo class switch recombination. This was monitored by staining the cells with fluorescently tagged anti-IgM antibody. As expected, the proportion of IgM+ cells was reduced at D4 compared to D0 (Fig. S2E), indicating that a subset of the cells had switched from producing the IgM B cell receptor isotype to the IgG1 isotype.

**Supplementary Figure Legends**

**Fig. S1**. Second derivative spectra of cells subjected to different treatments

(A) Average FTIR spectra and standard deviation for the three cell treatments; Control, TSA treated, and CIT treated (activated) as seen in Fig. 3F. (B) Second derivative of the average FTIR spectra seen in (A), calculated with 25-point quadratic Savitzky-Golay filter. (C) Amide I region: peptide C=O stretching. (D) Amide II region: primarily, NH bending. (E) Phosphate asymmetric stretching region: ν_as_(PO_2_^–^). (F) Phosphate symmetric stretching region: ν_s_(PO_2_^–^).

**Fig. S2**. Size, cell cycle progression and class switch recombination for primary B cells

(A) Flow cytometry data showing changes in size (FSC-A: forward scatter) and granularity (SSC-A: side scatter) for primary B cells at the timepoints D0, D1, and D2. (B) Nuclear size changes for primary B cells at the timepoints D0, D1, and D2. Minor axis (a) and major axis (B) as defined in Fig. 4C were measured for nuclear stained cells from each timepoint. (C) Cell cycle phase distribution for primary B cells at the timepoints D0, D1, and D2. (D) Quantified cell cycle phase distribution for primary B cells at the timepoints D0, D1, and D2. As the G0 stalled cells from the spleen (D0) start proliferating in culture (D1 and D2), the proportion of cells in S and G2/M phase increases. (E) Class switch recombination for primary B cells stained with an anti-IgM fluorescently tagged antibody, as measured by flow cytometry. Cells right after isolation from spleen (D0) were compared with cells cultured in medium with IL-4 and LPS for four days (D4). A reduction of IgM+ cells is seen over this time period.

**Fig. S3**. A cell translocating one of the microfluidic channels

(A) White-light image of the microfluidic chip showing the channels. (B) Cell ion the inlet approaching the channels. (C) The same cell translocating one of the microfluidic channels. (D) The same cell in the outlet. A further two cells on the left approaching the channels.
