# Supplementary figures and images for: Single Cell Imaging of Nuclear Architecture Changes
Source: Front Cell Dev Biol. 2019 Jul 24;7:141. doi: 10.3389/fcell.2019.00141 (PMC6668442; doi:10.3389/fcell.2019.00141)

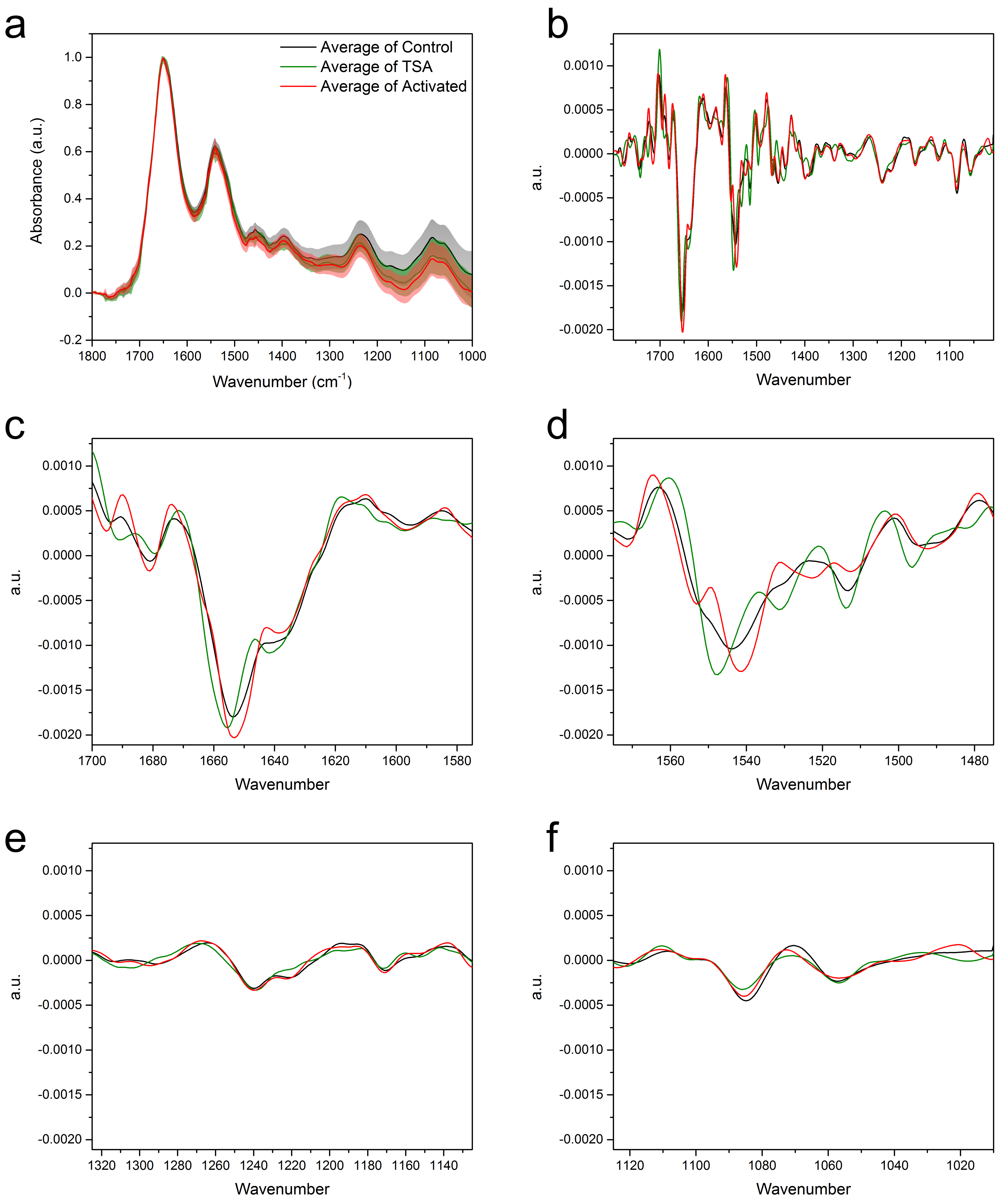

Supplement: Supplementary file 2 [file Image_1.TIF]

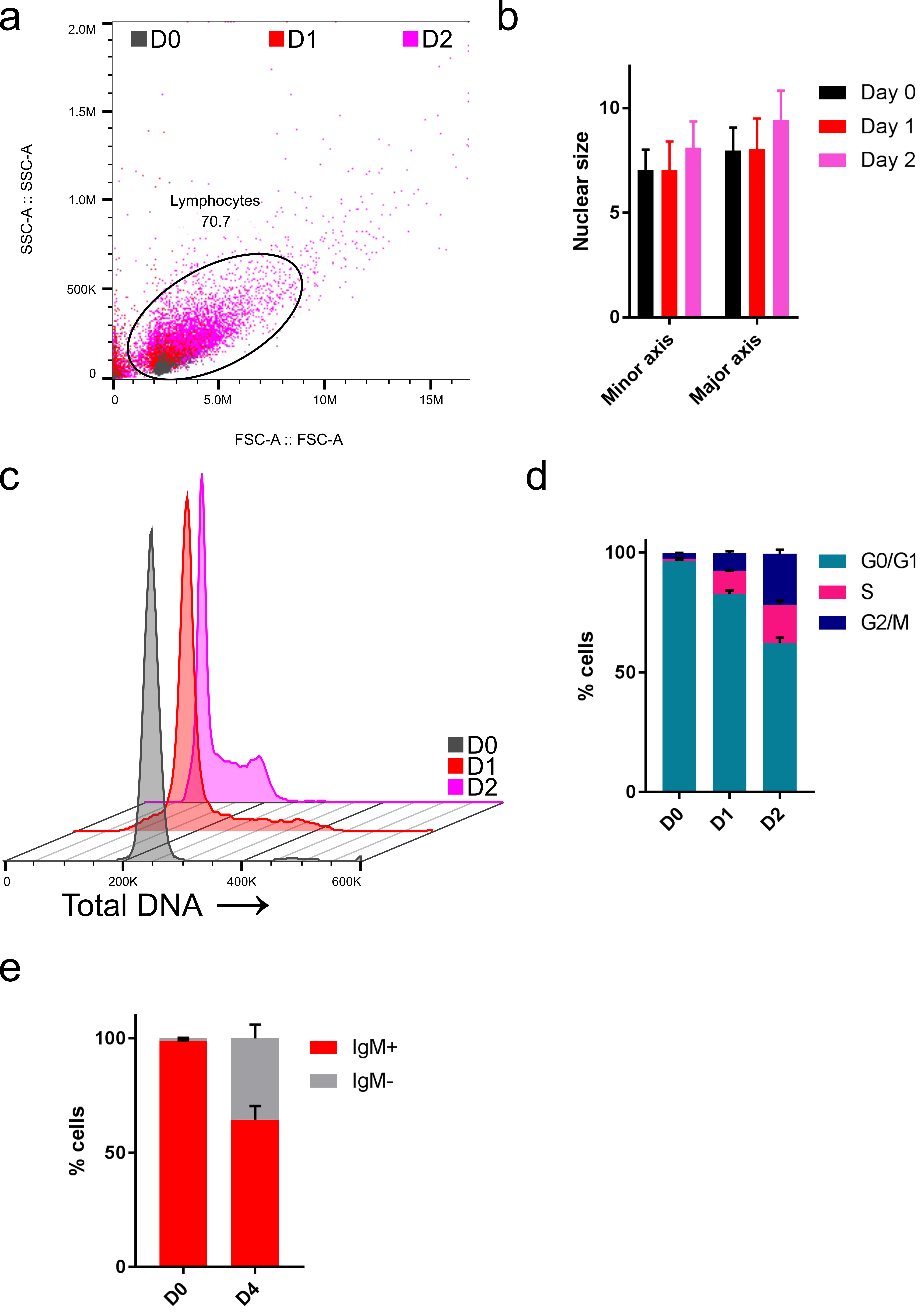

Supplement: Supplementary file 3 [file Image_2.TIF]

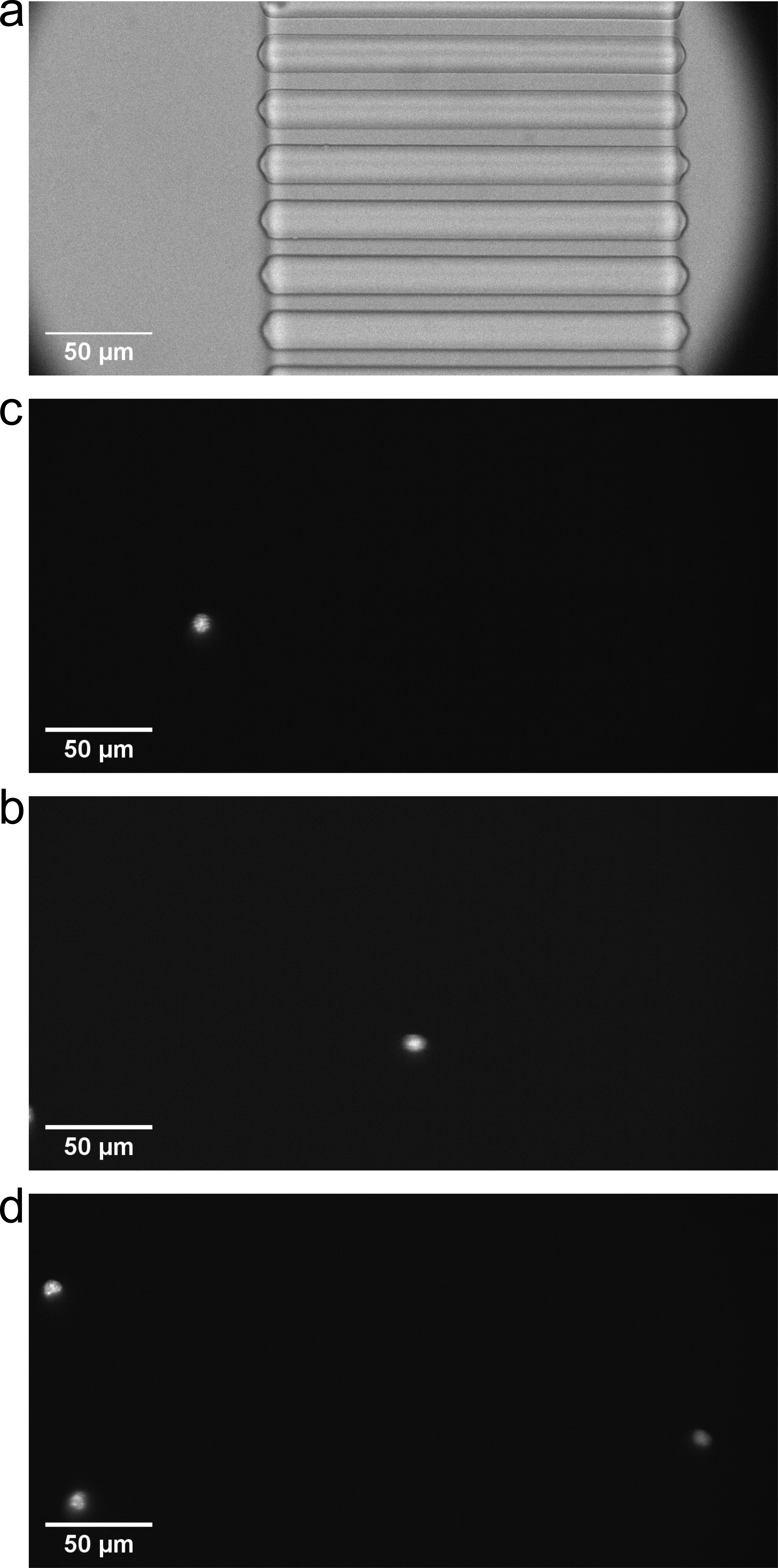

Supplement: Supplementary file 4 [file Image_3.JPEG]
